# Magnetoconductance Oscillations in Topological Crystalline Insulator Nanowires

**DOI:** 10.1021/acs.nanolett.5c02643

**Published:** 2025-10-07

**Authors:** Vince van de Sande, Mathijs G. C. Mientjes, Femke J. Witmans, Tim Hulsen, Xin Guan, Max S. M. Hoskam, Joost Ridderbos, Marcel A. Verheijen, Floris A. Zwanenburg, Alexander Brinkman, Fabrizio Nichele, Erik P. A. M. Bakkers

**Affiliations:** † Department of Applied Physics, 3169 Eindhoven University of Technology, P.O. Box 513, 5600 MB Eindhoven, The Netherlands; ‡ MESA+ Institute for Nanotechnology, University of Twente, P.O. Box 217, 7500 AE Enschede, The Netherlands; ¶ Eurofins Materials Science Eindhoven, 5656 AE Eindhoven, The Netherlands; § IBM Research Europe Zurich, 8803 Rüschlikon, Switzerland

**Keywords:** magnetoconductance, topological crystalline insulator, nanowires, oscillations, phase-coherent transport

## Abstract

Pb_1–*x*_Sn_*x*_Te is a topological crystalline insulator (TCI) hosting topological surface and hinge states protected by crystal mirror symmetries. The bulk carrier density can be reduced by tuning the Sn ratio *x*. Here, we perform low-temperature magnetotransport measurements on Pb_1–*x*_Sn_*x*_Te nanowires with varying *x* values grown by molecular beam epitaxy. We observe signatures of Aharonov–Bohm (AB)-type oscillations for 0.32 ≤ *x* ≤ 0.51, which coexist with aperiodic universal conductance fluctuations (UCFs) and are consistent with phase-coherent transport on the nanowire surface. We separately analyze the temperature dependence of the AB-type oscillations and UCFs. The oscillations give a phase coherence length of *l*_ϕ_ = 1.4 ± 0.2 μm at 80 mK, consistent with the ballistic transport regime. The UCF provides a significantly smaller *l*_ϕ_, consistent with a diffusive bulk transport channel, parallel to the ballistic surface. Our results indicate the presence of phase-coherent surface states on Pb_1–*x*_Sn_*x*_Te nanowires with 0.32 ≤ *x* ≤ 0.51.

## Graphical abstract

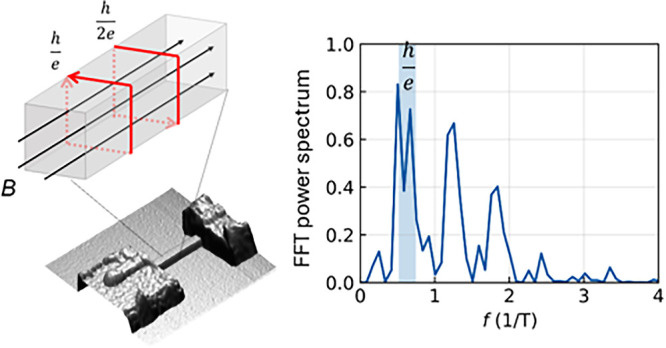

Topological crystalline insulators (TCIs) are a class of materials hosting gapless surface states, which are induced by band inversion in combination with strong spin–orbit coupling (SOC) and are topologically protected by symmetries of the crystal lattice.^1,2^ TCI surface states have properties similar to surface states of topological insulators (TIs), such as spin-momentum locking and a Dirac-like band structure (although quadratic band structures have also been proposed^2^).^3,4^ Furthermore, crystal symmetry protection in TCIs enables distinct features, such as higher order topological states that can coexist with surface states. Breaking these symmetries through strain, electric fields, or ferroelectric lattice distortions enables tuning of topological states and control over topological phase transitions.^5–12^

Owing to its simple rock-salt cubic crystal structure, the TCI SnTe is a promising material platform to study topological states protected by mirror symmetry.^5^ Theoretical predictions have indicated that SnTe nanowires with {100} facets host hinge and corner states and, when proximitized by a superconductor, may host localized Majorana zero modes.^13^ Moreover, under rhombohedral lattice distortion, SnTe nanowires acquire the helical higher-order topological insulator (HOTI) phase.^10^ The surface Dirac band structure has been directly observed using angle-resolved photoemission spectroscopy (ARPES).^14^ Furthermore, Fabry–Pérot interference of surface states has been observed in SnTe nanowires, indicating ballistic surface transport.^15^ A recent theoretical study has reported that the surface states on {100} facets of SnTe nanowires are extended across their surfaces, allowing for the observation of Aharonov–Bohm (AB)-type oscillations.^16^ Under application of a parallel magnetic field, the conductance oscillates with the period Φ_0_ = *h*/*e*, the magnetic flux quantum. These oscillations arise from the quantization of surface state momentum perpendicular to the nanowire and have provided a means to isolate contributions of the surface states, even in the presence of a conducting bulk.^17,18^ We note that AB-type oscillations are not a direct signature of topological protection but rather indicate phase-coherent transport.^19^ Experimental studies have reported AB-type oscillations in TI nanowires, including Sb_2_Te_3_,^20^ Bi_2_Te_3_,^21–24^ and Bi_2_Se_3_,^25–28^ and even in SnTe nanowires.^29^

Although SnTe exhibits fascinating physical properties, electronic transport studies have been challenging due to the formation of Sn vacancies, which act as acceptors.^30–32^ This has resulted in a highly p-doped semiconductor with a bulk carrier density *p*_b_ of 10^20^–10^21^ cm^–3^.^33,34^ The transport signal is dominated by charge carriers from the bulk, masking the contribution of the topological surface states. Pb_1–*x*_Sn_*x*_Te has a significant advantage over the binary SnTe, as *p*_b_ can be reduced by several orders of magnitude by decreasing *x*, while retaining the band inversion until *x* ≈ 0.3, below which the band gap is trivial.^35–37^ Even though Pb_1–*x*_Sn_*x*_Te is more suited for investigating surface state transport, few studies have reported experimental transport results.^34,38–40^ Pb_1–*x*_Sn_*x*_Te nanowires are particularly promising due to their large surface–bulk ratio,^41^ but only one study has reported magnetotransport results, reporting a weak antilocalization (WAL) effect for *x* = 0.5 and *x* = 0.8, a signature of strong SOC.^42^ Aharonov–Bohm oscillations in Pb_1–*x*_Sn_*x*_Te nanowires have not yet been observed.

Here, we grow single-crystalline Pb_1–*x*_Sn_*x*_Te nanowires with well-defined {100} facets. We tune the nanowire composition from the predicted trivial to the topological regime and study the magnetoconductance across the phase transition. We identify periodic oscillations for 0.32 ≤ *x* ≤ 0.51, consistent with Aharonov–Bohm-type oscillations from phase-coherent surface states.

Pb_1–*x*_Sn_*x*_Te nanowires were grown using the vapor–liquid–solid mechanism in molecular beam epitaxy (MBE). The composition of the nanowires was controlled by adjusting the ratio of the Pb and Sn fluxes during growth. A detailed discussion of the sample preparation and growth scheme can be found in Section 1 of the Supporting Information (SI). Figure 1a shows a nanowire in bright-field transmission electron microscopy (BF-TEM), with its associated diffraction pattern in Figure 1b. The nanowires are single-crystalline with a rock salt crystal structure and are terminated by atomically flat facets of the {100} family, covered with approximately 3 nm native oxide. The composition of the nanowires was analyzed using energy dispersive X-ray (EDX) spectroscopy, and the quantification procedure has been described in detail in ref 41. A typical position-dependent EDX measurement is depicted in Figure 1c. The composition is constant over the entire nanowire length. More information is presented in Section 2 of the SI.

**Figure 1:**
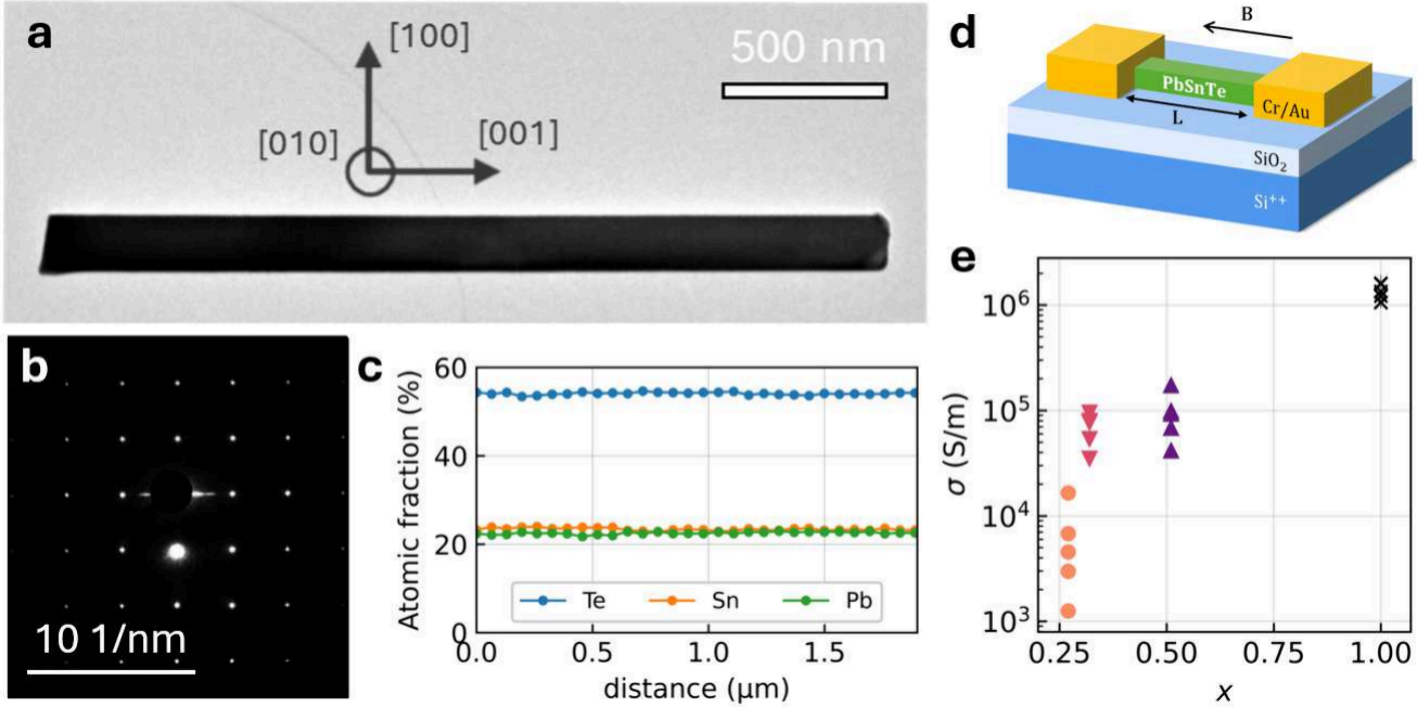
a) BF-TEM image of a Pb_1–*x*_Sn_*x*_Te nanowire with *x* = 0.51, with arrows indicating the crystal directions. b) Electron diffraction pattern associated with a). c) EDX compositional line profile of a Pb_1–*x*_Sn_*x*_Te nanowire with *x* = 0.51. The atomic fractions of Te, Pb, and Sn are plotted as a function of distance along the nanowire. The growth direction is from left to right. d) Schematic representation of a nanowire device. e) Conductivity σ of the devices plotted as a function of *x*.

Nanowires were transferred onto highly doped Si (100) substrates covered with SiO_2_ and prepatterned with Cr/Au markers and contact pads. To form ohmic contacts, the native oxide layer on the nanowire surface was removed with Ar ion milling. Cr/Au contacts were evaporated without breaking the vacuum to prevent the reformation of native oxide after the milling step. Additional fabrication details and cross-sectional TEM images of the contact interface confirming that there is no remaining oxide at the interface are presented in Section 3 of the SI.

We investigated multiple Pb_1–*x*_Sn_*x*_Te nanowire devices with *x* = 0.27, 0.32, 0.51, and 1. The nanowires varied in dimensions, with lengths of 1–3 μm and diameters of 40–200 nm. The width *W* and height *H* of the nanowires were determined using atomic force microscopy (AFM), of which the analysis is presented in Section 4 of the SI. We fabricated and measured devices with two (see the schematic in Figure 1d) or four contacts depending on the length of the individual nanowires. The conductance *G* was measured by applying a voltage or current bias, depending on the conductance of each device. Devices A1 and A2 were measured using a DC bias method, the details of which are reported in Section 5 of the SI. All other devices were measured using a low-frequency lock-in method, with a typical AC bias of 10 nA. An overview of the devices is presented in Section 6 of the SI.

Figure 1e shows the conductivity σ = *lA*/*R*, where *l* is the channel length, *A* = (*H* – 6 nm)­(*W* – 6 nm) is the cross-sectional area of the nanowire corrected for a 3 nm native oxide layer, and *R* is the resistance at zero magnetic field. For the devices with two contacts, we subtracted a series resistance, *R*_c_, to account for the measurement setup. The conductivity is observed to decrease by 3 orders of magnitude between nanowires with *x* = 1 and *x* = 0.27. This is consistent with the expected decrease in bulk carrier density *p*_b_, since σ ∝ *p*_b_, indicating that the formation of electrically active Sn vacancies is largely suppressed by reducing *x*.^34^

The magnetoconductance of the devices was measured in a dilution refrigerator with the mixing chamber temperature *T* kept at 80 mK to reduce the effect of heating during magnetic field sweeps. The in-plane magnetic field *B*_*z*_ was applied by using a superconducting vector magnet. We selected nanowires that were approximately aligned along the direction of *B*_*z*_, but their random deposition on the substrate resulted in some variation in the angle with respect to *B*_*z*_.

Figure 2 shows the magnetoconductance *G* of four nanowires with varying *x*, together with the corresponding Fourier power spectra. The insets show AFM topography images of the corresponding devices. A large variation in the average conductance for varying *x* is observed, with almost 3 orders of magnitude difference in *G*(*B*_*z*_ = 0) between *x* = 1 (SnTe) and *x* = 0.27, in agreement with Figure 1e. For *x* = 1 in Figure 2a, *G* is dominated by the slowly varying background due to positive magnetoresistance, commonly observed in metals and doped semiconductors.^43^ In contrast, for *x* = 0.51 and *x* = 0.32 in Figure 2b,c, *G* is dominated by pronounced fluctuations. Lastly, for the device with *x* = 0.27 in Figure 2d, less pronounced fluctuations on top of a slowly varying background are observed.

**Figure 2:**
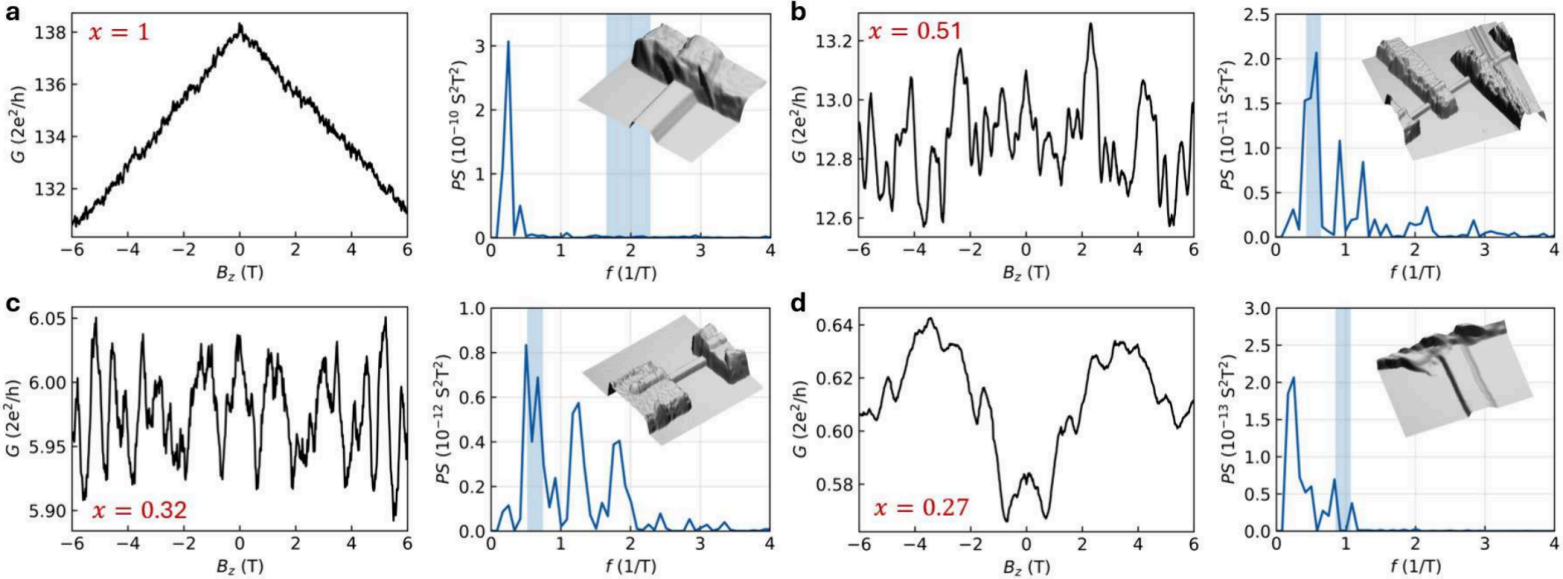
Magnetoconductance traces and fast Fourier transforms of Pb_1–*x*_Sn_*x*_Te nanowire devices with a) *x* = 1 (device F7), b) *x* = 0.51 (device B1), c) *x* = 0.32 (device A1), and d) *x* = 0.27 (device F11). The magnetoconductance traces display the conductance *G* as a function of the magnetic field in the *z*-direction, *B*_*z*_, and were taken at *T* = 80 mK. The Fourier transforms display the power spectrum *PS* in *S* ^2^ *T* ^2^ as a function of the frequency *f* in 1/*T*. The blue regions indicate the expected frequency range for Φ_0_-periodic oscillations, determined by the nanowire dimensions and associated uncertainties. The insets show AFM images of the corresponding devices.

The data were analyzed in more detail by calculating the FFT spectra, the details of which are reported in Section 7 of the SI, along with an overview of the Fourier spectra of all devices. We calculated the magnetic flux ϕ = *AB*_*z*_ cos­(θ) through the cross-sectional area of the nanowire, where θ is the angle of the nanowire with respect to *B*_*z*_. The period for the AB-type oscillations becomes *B*_0_ = Φ_0_/*A*, with Φ_0_ = *h*/*e* being the magnetic flux quantum. The frequency corresponding to a single flux quantum Φ_0_ threading through the cross-sectional area of the nanowire, defined as *f*_0_ = 1/*B*_0_, is colored light blue.

For *x* = 1 in Figure 2a, the FFT spectrum is dominated by a low-frequency peak, which corresponds to the residual slowly varying background after subtracting a fourth-order polynomial fit. No peaks appear at the frequency corresponding to the cross-sectional area of the nanowire. For *x* = 0.51 and *x* = 0.32 in Figures 2b,c, the highest peak in the spectrum overlaps with the frequencies indicated in light blue, consistent with periodic oscillations in *G* due to the interference of phase-coherent charge carriers encircling the nanowire. For *x* = 0.32, two additional peaks match well with the higher harmonics *h*/2*e* and *h*/3*e*, while for *x* = 0.51, higher frequency peaks are observed that do not align well with the expected harmonics. Peaks at the expected frequencies are observed for four out of five devices for *x* = 0.32 and only two out of five for *x* = 0.51. For *x* = 0.27 in Figure 2d, the spectrum is dominated by a low-frequency peak similar to that in Figure 2a, and at the frequency indicated in light blue, no peak is observed. This aligns with the expectation that Pb_1–*x*_Sn_*x*_Te with *x* = 0.27 is a trivial semiconductor without surface states.^37^

To further investigate the periodic oscillations for *x* = 0.32 and *x* = 0.51, we measured the magnetoconductance as a function of *T*. Figure 3a shows the FFT power spectrum for *x* = 0.32 as a function of *T* with the AB frequency indicated in light gray. The height of the peak decreases with an increasing temperature, corresponding to a decreasing oscillation amplitude. We extracted the amplitude *A* of the peaks *h*/*e* and *h*/2*e* by applying a Gaussian filter and integrating to obtain the area below the peak; see Section 7 in the SI. Figure 3b shows the normalized amplitude *A*/*A*_0_ as a function of temperature, which increases drastically below *T* ≈ 1 K. Above this temperature, the peak becomes too small to be reliably resolved from other features in the FFT spectrum. We fitted a model to the low-temperature part which has the form

1 $$A_{h/ne}=A_{0}\cdot e^{-nL/l_{\phi }}$$

Here *n* is the winding number for *h*/*ne* oscillations, *L* is the nanowire circumference and *l*_ϕ_ = *C*·*T* ^–*m*^ is the phase coherence length, where *A*_0_ and *C* are fitting parameters and *m* = 0.5 for diffusive transport or *m* = 1 for ballistic transport.^26,44^ The data fit best with *m* = 1 for both *h*/*e* and *h*/2*e*, indicating that the relevant transport regime is ballistic. We therefore assume that the decoherence mechanism is weak coupling of ballistic surface states to fluctuations in the environment,^45^ similar to TI nanowires.^23,24,26^ From the fit of the *h*/*e* data, we obtain *l*_ϕ_ as a function of temperature with *l*_ϕ_ = 1.4 ± 0.2 μm at *T* = 80 mK, as shown in Figure 3c. The same analysis for *x* = 0.51 is shown in Figures 3d–f. The low-temperature data fit best with *m* = 1, indicating ballistic transport, similar to *x* = 0.32, but with a smaller coherence length *l*_ϕ_ = 640 ± 80 nm at *T* = 80 mK. More details on the fitting are reported in Section 8 of the SI.

**Figure 3:**
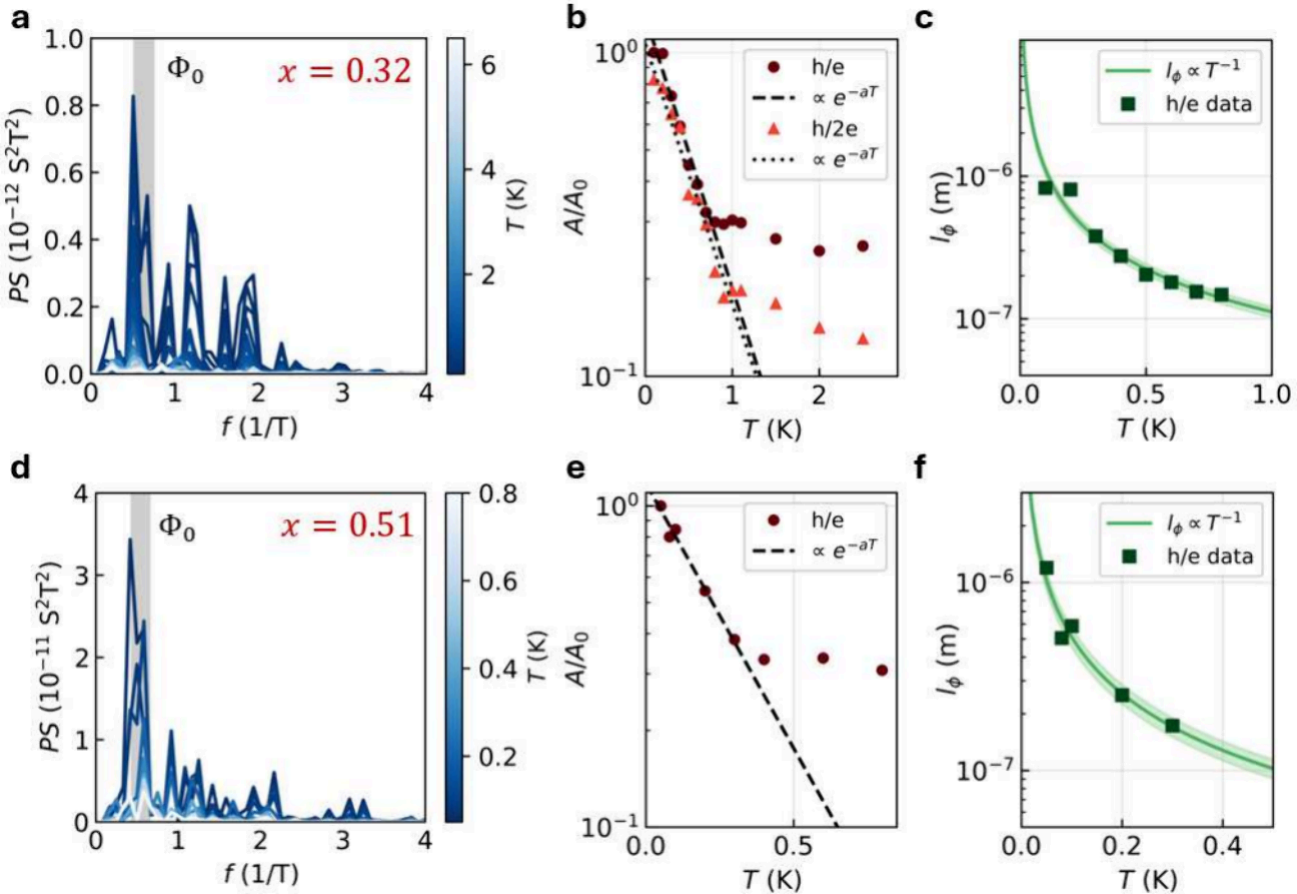
Temperature dependence of the AB-type oscillations for *x* = 0.32 (device A1) and *x* = 0.51 (device B1). a) FFT power spectrum of device A1 as a function of *T*, with the *h*/*e* frequency *f*_0_ colored gray and indicated with Φ_0_. b) Integrated amplitude of the *h*/*e* and *h*/2*e* peaks in a) as a function of *T*, fitted below *T* ≈ 1 K with a function of the form *e* ^–*aT*^. c) *l*_ϕ_ as a function of *T*, calculated from the fit in b). The green line indicates an exponential decrease according to *l*_ϕ_ ∝ *T* ^–1^, with uncertainty from the fit. d–f) Same as a–c) but for device B1 (*x* = 0.51).

To explain the longer phase coherence length for *x* = 0.32 compared to *x* = 0.51, we hypothesize that bulk properties may play a significant role. Although spin-momentum locking drastically decreases the chance that surface carriers scatter into other surface states, this does not prevent them from scattering into bulk states.^46^ For higher *x*, the Fermi level lies deeper in the valence band, and therefore, more bulk states become available for the surface states to scatter with. Moreover, the ratio of the total amount of surface carriers to the total amount of bulk carriers depends strongly on *x*. From Figure 1e, we assume that the bulk hole density *p*_b_ of Pb_1–*x*_Sn_*x*_Te increases significantly for *x* > 0.27, similar to calculations from Hall effect measurements on epilayers of Pb_1–*x*_Sn_*x*_Te grown with MBE.^34^ Therefore, we assumed similar values for the bulk carrier density. We assume a constant surface state carrier density *p*_*s*_ ≈ 5 × 10^12^ cm^–2^, calculated from the structure of the surface band of a thin film of SnTe measured in ARPES,^47^ independent of the bulk.^48^ Although the surface state carrier density generally depends on the position of the Fermi level relative to the Dirac point, we do not account for this variation in our simplified calculation. For a nanowire with typical diameter *D* = 100 nm, the contribution of the surface states to the total number of charge carriers is *N*_ss_ ≈ 1% for *x* = 1, *N*_ss_ ≈ 17% for *x* = 0.51, and *N*_ss_ ≈ 50% for *x* = 0.32. More details are reported in Section 9 of the SI. So, even though surface states are present in SnTe, the conductance is likely to be dominated by bulk carriers.

We now present an analysis of the full set of measured devices. Specifically, we computed the integrated peak amplitude at the frequencies *h*/*e*, *h*/2*e*, and *h*/3*e*, denoted *U*, and the total integrated spectrum, *U*_tot_, the details of which are reported in Section 7 of the SI. The quantity *U*/*U*_tot_ provides the relative contribution of these specific frequencies compared with the overall signal. Figure 4a shows the *U*/*U*_tot_ as a function of *x*. For 4 devices with *x* = 0.32, and 2 devices with *x* = 0.51, *U*/*U*_tot_ × 100 > 10%. These are the devices that showed a peak at the Aharonov–Bohm frequency in the FFT.

**Figure 4:**
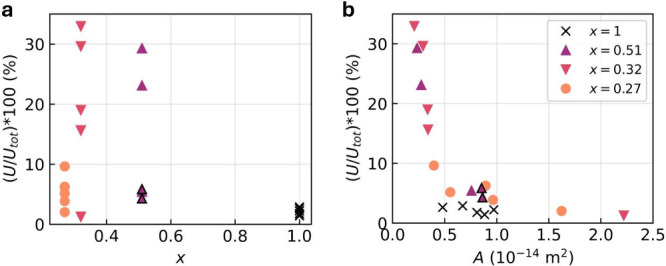
Integrated peak amplitudes of the frequencies *h*/*e*, *h*/2*e*, and *h*/3*e* divided by the total FFT spectrum amplitude *U*/*U*_tot_ × 100 a) as a function of *x* and b) as a function of the nanowire cross-sectional area *A* for different values of *x*. All measurements were performed at *T* = 80 mK, except for devices B4 and B5 (*x* = 0.51), which were measured at *T* = 100 mK (highlighted in the figure by black borders around the corresponding markers).

Figure 4b shows that there is a significant dependence of the oscillation amplitude on the cross-sectional area *A*. Specifically, *U*/*U*_tot_ increases significantly for *A* < 0.5 × 10^–14^ m^2^, which corresponds to a diameter *D* ≈ 70 nm for a square cross section. We suggest possible reasons for this behavior in the following. Because of the finite coherence length, the charge carriers on the surface are more likely to remain phase-coherent for thin nanowires with a shorter circumference. Moreover, the mean level spacing of the surface states Δ scales inversely with *D* according to Δ = *ℏv*_f_π/2*D* with *v*_f_ the Fermi velocity, and therefore averaging over energy levels is reduced for wires with smaller diameter, meaning that AB-type oscillations are more easily washed out for large diameter wires.^17,49^ Lastly, the higher surface-to-bulk ratio of thin nanowires leads to a significantly enhanced contribution of the surface states to the total signal, as mentioned before. The ratio of surface carriers *N*_s_ to bulk carriers *N*_b_ scales inversely to the diameter of the nanowire: *N*_s_/*N*_b_ ∝ 1/*D*. Possibly, AB-type oscillations might become visible for SnTe nanowires with even smaller diameters despite the large bulk carrier density. We note that if the cross section becomes too small, the ±6 T range of the magnetic field becomes limiting for the number of visible oscillations. The relevant peak in the FFT corresponding to the nanowire circumference will then overlap with the residual peak corresponding to the slowly varying background, making a distinction difficult. We therefore excluded nanowires with *A* < 2 × 10^–15^ m^2^.

Finally, we analyzed the aperiodic conductance fluctuations, which are superimposed on periodic AB-type oscillations in the magnetoconductance. The temperature dependence of these fluctuations provides another way to estimate the coherence length. Figure 5a shows the inverse fast Fourier transform (IFFT) of the *h*/*e* peak for device A1 with *x* = 0.32 in Figure 3a. Figure 5b shows the data after subtracting the IFFT data including the higher harmonics *h*/2*e* and *h*/3*e*, which then correspond to the aperiodic fluctuations. From this, we calculated the autocorrelation function *F*(Δ*B*_*z*_) = ⟨*δG*(*B*_*z*_ + Δ*B*_*z*_)*δG*(*B*_*z*_)⟩ for varying *T* in Figure 5c. We then used the magnetic field at half-maximum of the autocorrelation function *F*(*B*_c_) = 1/2*F*(0), with *B*_c_ the correlation field, to compute *l*_ϕ_ = γΦ_0_/*B*_c_ *d*, where γ = 0.42 for *l*_ϕ_ smaller than the thermal length^50,51^ and *d* determined from the dimensions of the nanowire. Additionally, a purely geometric correction was applied to correct for the aspect ratio and angle, with respect to *B*_*z*_. More details are reported in Section 10 of the SI.

**Figure 5:**
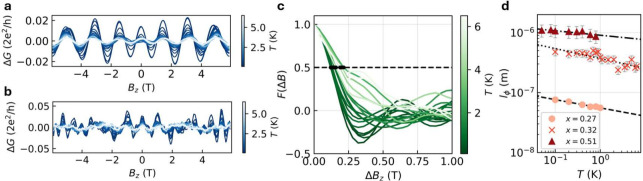
a) IFFT of the *h*/*e* peak for different temperatures for *x* = 0.32 (device A1). b) The magnetoconductance for different temperatures of device A1, with the IFFT of the *h*/*e*, *h*/2*e*, and *h*/3*e* peaks subtracted from the data. c) Autocorrelation function *F*(Δ*B*) of the data in b). The black dots indicate the correlation field *B*_c_. d) Coherence length *l*_ϕ_ versus *T* for *x* = 0.27, 0.32, and 0.51. Values shown were corrected for device angle and aspect ratio; see Section 10 of the SI. The data are fitted with a fit of the form *l*_ϕ_ ∝ *T* ^–*m*^, where *m* = 0.14, 0.17, 0.08 for *x* = 0.27, 0.32, 0.51, respectively.

Figure 5d shows *l*_ϕ_ as a function of *T*, for devices B1 (*x* = 0.51), A1 (*x* = 0.32), and F11 (*x* = 0.27). The coherence length *l*_ϕ_ decreases with decreasing *x* and is larger than the diameter of the nanowire for all three devices at low temperature. For *x* = 0.32, *l*_ϕ_ ≈ 560 nm at *T* = 80 mK, which is significantly shorter than *l*_ϕ_ determined from the temperature dependence of the AB effect in Figure 3c. Moreover, *l*_ϕ_ for *x* = 0.51 is larger than that for *x* = 0.32, despite the analysis in Figures 3c,f, which indicated that nanowires with *x* = 0.32 had a significantly larger *l*_ϕ_ than *x* = 0.51. We therefore conclude that the conductance fluctuations arise from diffusive bulk states, which form a parallel transport channel to the surface states.

The data in Figure 5d were fitted using a function of the form *l*_ϕ_ ∝ *T* ^–*m*^. For Nyquist dephasing in a quasi-1D system, the expected temperature dependence of *l*_ϕ_ follows *T* ^–1/3^.^52,53^ The decay of *T* ^–0.17^ for *x* = 0.32 is lower than *T* ^–1/3^, and we find an even smaller temperature dependence for *x* = 0.27 with *T* ^–0.14^ and *x* = 0.51 with *T* ^–0.08^. This deviation from theoretical expectations is similar to reported values on quasi-1D nanowires, e.g., InN and Sb_2_Te_3_.^54–56^ The observed increase in the coherence length as a function of *x* may be attributed to enhanced screening of electron–electron interactions as a result of the increased bulk carrier density.

In conclusion, we conducted an extensive magnetoconductance study of Pb_1–*x*_Sn_*x*_Te nanowire devices with varying Sn-fraction *x* and observed periodic oscillations for 0.32 ≤ *x* ≤ 0.51, at a frequency corresponding to the circumference of the nanowire. We attribute this observation to Aharonov–Bohm-type oscillations facilitated by phase-coherent surface states. From the temperature dependence of the oscillations, we conclude that the transport regime is ballistic. For a nanowire with *x* = 0.32, we estimate a coherence length for the surface states that is significantly longer than that of the bulk carriers. Nevertheless, the topological nature of the Aharonov–Bohm-type oscillations remains unclear and requires further experimental investigation. Our findings highlight Pb_1–*x*_Sn_*x*_Te as a promising platform for electronic transport studies of phase-coherent surface states in a topological crystalline insulator. The observation of ballistic surface transport despite residual bulk conductance provides a path for exploring topological phenomena in this system such as higher-order topological states and topological superconductivity.

## Supplementary Material



## Notes

Additional information on the growth, nanowire composition analysis, TEM/EDX data, electronic transport measurements, and data analysis, additional AFM data and FFT spectra, temperature dependence fits, surface–bulk ratio calculation, and discussion on UCF (PDF)
