# Supplementary material for: Magnetoconductance Oscillations in Topological Crystalline Insulator Nanowires
Source: Nano Lett. 2025 Oct 7;25(41):14793–9. doi: 10.1021/acs.nanolett.5c02643 (PMC12532288; doi:10.1021/acs.nanolett.5c02643)
Supplement: Supplementary file 1 [file nl5c02643_si_001.pdf]

Supporting Information

Magnetoconductance oscillations in topological crystalline insulator nanowires

Vince van de Sande[1], Mathijs G.C. Mientjes[1], Femke J. Witmans[2], Tim Hulsen[1], Xin Guan[1], Max S.M. Hoskam[1], Joost Ridderbos[2], Marcel A. Verheijen[1,3], Floris A. Zwanenburg[2], Alexander Brinkman[2], Fabrizio Nichele[1,4], Erik P.A.M. Bakkers[1*]

[1] *Department of Applied Physics, Eindhoven University of Technology, P.O. Box 513, 5600 MB Eindhoven, The Netherlands*
[2] *MESA+ Institute for Nanotechnology, University of Twente, P.O. Box 217, 7500 AE Enschede, The Netherlands*
[3] *Eurofins Materials Science Eindhoven, 5656 AE Eindhoven, The Netherlands*
[4] *IBM Research Europe Zurich, 8803 Rüschlikon, Switzerland*

September 4, 2025

*Corresponding author: e.p.a.m.bakkers@tue.nl

1 Nanowire growth

The $Pb_{1-x}Sn_xTe$ nanowires were grown on ⟨100⟩-oriented Si substrates with a 20 nm SiN mask. Holes were patterned in this mask with varying hole size (20-100 nm) and pitch (500-2000 nm) using electron beam lithography followed by a BHF dip to etch the holes. We found that the addition of the SiN mask promoted growth within the holes while impeding growth on the mask. This should allow for better control of the nanowire morphology. Prior to growth, 10 nm of Au was deposited within the holes. The Au droplets acted as catalyst particles during the growth, allowing for a higher aspect ratio (ratio of length to diameter) of the nanowires. Before the growth phase, the samples were degassed at a temperature of 300 °C for approximately one hour in a high vacuum environment. The samples were then transferred to the growth chamber without breaking the vacuum. During growth, the substrate temperature was monitored using the band edge absorption (BandiT) of a GaAs reference substrate and was kept constant at 359 °C. Material was deposited from Pb and Sn effusion cells and a Te cracker cell. The fluxes were measured in beam equivalent pressure (BEP) using a beam flux monitor before the growth. Before the growth phase, a 4 minute pre-deposition step of only the group IV material was added, as this was found to increase the NW yield. The growth phase consisted of a 220 minute deposition of Pb, Sn and Te. The total flux of material on the substrate is denoted as F_{tot}. In this work, F_{tot} was kept fixed at $2x10^{-7}$ BEP and the group IV to group VI flux ratio was kept at 0.7. The Sn-fraction was controlled by varying the ratio of the Pb and Sn flux. We found that nanowires could be grown using this method over the entire composition regime (not shown in this work).

2 Nanowire composition

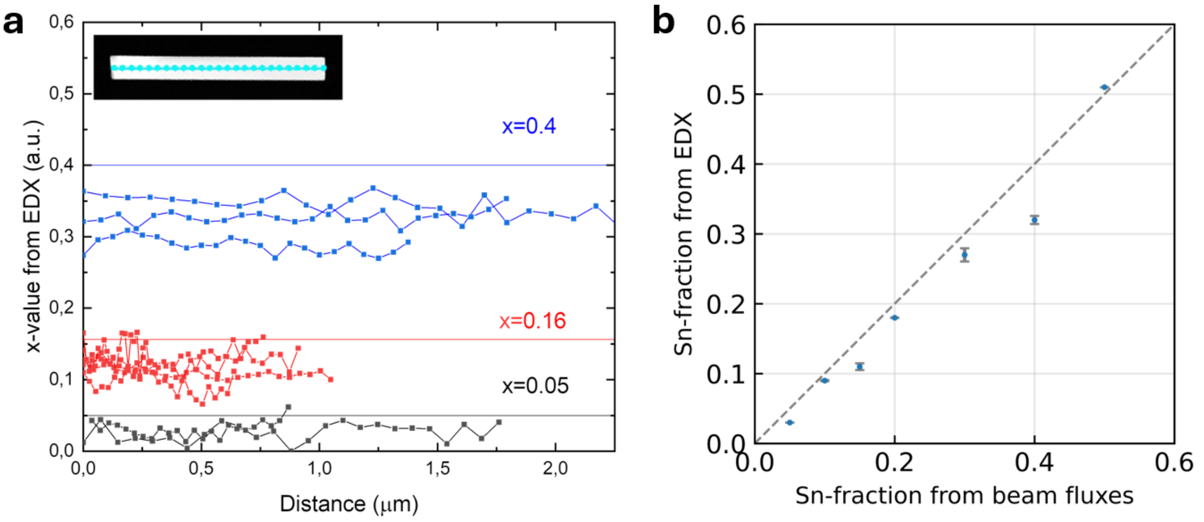

Figure 1: Composition analysis of $Pb_{1-x}Sn_xTe$ nanowires. The quantification procedure is described in detail in [1]. a) The Sn fraction x, as determined by EDX, along the length of several nanowires (see inset) from growth runs with an input Sn-fraction of $x = 0.05$ (black), $x = 0.16$ (red) and $x = 0.4$ (blue). b) The average Sn-fraction of multiple NWs within one growth run for multiple growth runs for varying input Sn-fraction. The black line depicts the line $X = Y$.

3 Contact fabrication

To establish Ohmic contacts, the work function of the nanowire must be aligned with that of the metal contacts. For p-type doping, which was observed for all nanowires, the condition for Ohmic contact formation is $\phi_m \geq (\chi_{nw} + E_g)$, with ϕ_m the metal work funtion, χ_{nw} the electron affinity of the nanowire and E_g the band gap of the nanowire. In this study, Au was chosen as the contact material because of its large work function $\phi_m = 5.1$, which fulfills the above requirement for p-type $Pb_{1-x}Sn_xTe$, regardless of

x [2]. It is necessary to use a thin Cr sticking layer to promote the adhesion of Au to the nanowire surface. Cr has a lower work function of $\phi = 4.5$ eV, such that Schottky barriers might be formed depending on the nanowire composition [3]. For nanowires with $x \geq 0.32$, Ohmic contacts with low resistance were consistently observed. For $x = 0.27$, we observed that a 10 nm layer of Cr resulted in a substantial contact resistance and instability of the devices, indicating Schottky barrier formation at the Cr-nanowire interface. To address this issue, a thinner Cr layer of 4-5 nm was used to facilitate tunneling through the Schottky barriers, which substantially reduced the contact resistance and improved the stability of the devices with $x = 0.27$.

To verify the quality of the interface between the Cr/Au and the $Pb_{1-x}Sn_xTe$ nanowire after fabrication, a cross-section of device B1 ($x = 0.51$) along the axial direction was made using focused ion beam (FIB) sample preparation, see figure 2a. High-angle annular dark field (HAADF) STEM data of this cross-section is shown in Figure 2b. The dark region at the bottom is the substrate and the lighter region in the middle is the nanowire with Cr/Au contacts on top. Figure 2c shows a bright-field (BF) STEM image zoomed in on the interface between the nanowire (NW) and the metal contact, close to the top of the nanowire (left-most part in Figure 2b). A crystalline layer of Cr is present directly on top of the nanowire. Figure 2 shows a high-resolution (HR) TEM image zoomed in on a different part of the contact. Lattice fringes are visible in all three materials. No amorphous interface layer is present, indicating that the native oxide layer of the nanowire has been removed in the fabrication process.

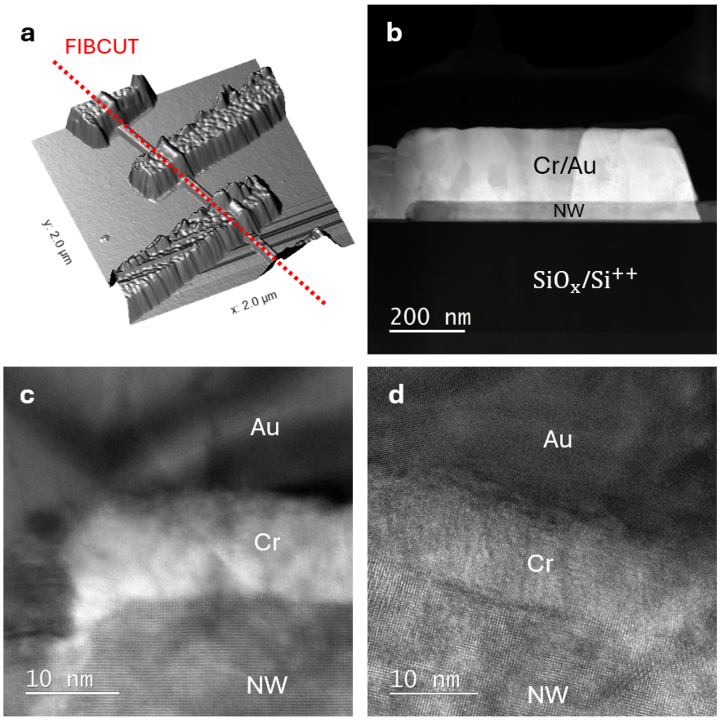

Figure 2: TEM data on device B1 showing the absence of an amorphous interface layer between the Cr/Au lead and the $Pb_{1-x}Sn_xTe$ nanowire ($x = 0.51$). a) AFM image, with the direction of the FIB cut indicated with a red dotted line. b) HAADF STEM image. c) BF STEM image. d) HR TEM image.

4 Atomic force microscopy

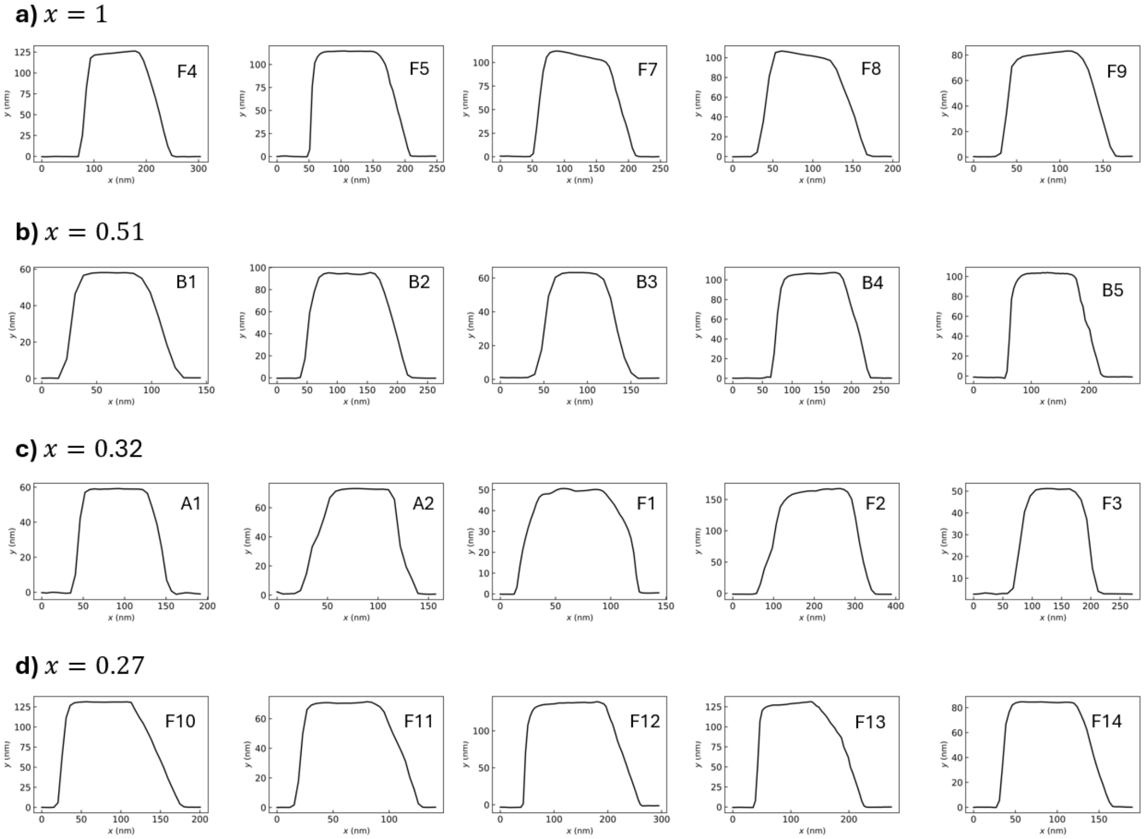

Figure 3: Overview of AFM cross-sections of the measured $Pb_{1-x}Sn_xTe$ nanowire devices with a) $x = 1$, b) $x = 0.51$, c) $x = 0.32$, d) $x = 0.27$. The insets display the corresponding device identifiers.

5 Electronic transport measurements

All devices, with the exception of A1 and A2, were measured at the Eindhoven University of Technology in a Bluefors dilution refrigerator with base mixing chamber temperature $T < 7$ mK. The temperature was kept at $T = 80$ mK for all measurements, unless stated otherwise. We measured the magnetoconductance using low-frequency lock-in methods, with a frequency between 50 and 500 Hz. We applied a current bias of typically 10 nA for devices with $x = 0.32$ or $x = 0.51$, and 20 nA for devices with $x = 1$. For $x = 0.27$, a voltage bias of typically 20 µV was applied, due to the larger resistance of these devices. Devices B1, F4 and F8 were measured using 4 electrodes, with the bias applied on the outer electrodes and the voltage measured over the inner electrodes. All other devices were measured using two electrodes. Current bias was applied by adding a 10 MΩ resistor in series with the lock-in amplifier. We used a SP983c I-V amplifier and a SP1004 differential amplifier from Basel Precision Instruments for voltage- and current-biased measurements, respectively. The magnetoconductance data shown in Figures 2a and 2b of the main text represent the average of two magnetic field sweeps ranging from -6T to +6T and back. For Figure 2d, the data was averaged over four such sweeps.

Devices A1 and A2 were measured at the University of Twente in an Oxford dilution refrigerator with base $T \approx 20$ mK. The magnetoconductance traces of devices A1 (presented in the main text in Figure 2c) and A2 (presented in Figure 6) were taken as line cuts of the 2D maps shown in Figure 4 at $V_{sd} \approx 0$ V. Here, the current I_{D} was measured as a function of DC bias voltage V_{SD} and in-plane magnetic field B_{z}. A moving average was applied to the data along B_z and the differential conductance was computed using $\delta I_{\mathrm{D}}/\delta V_{\mathrm{SD}}$.

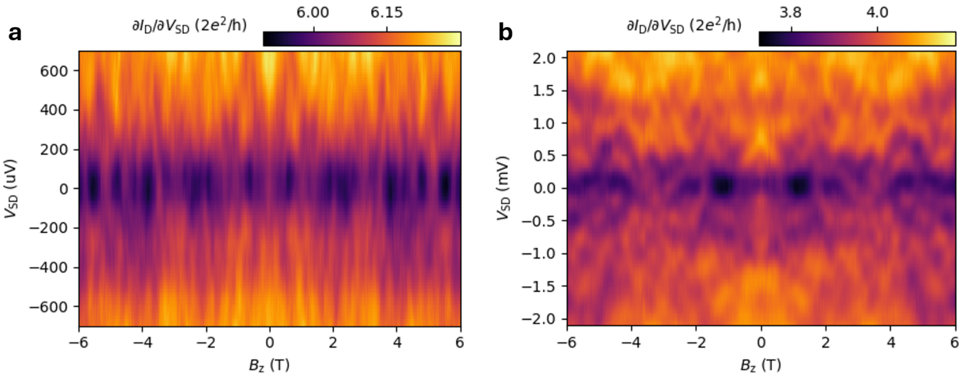

Figure 4: Differential conductance $\delta I_D/V_{SD}$ as a function of bias voltage V_{SD} and magnetic field B_Z at T = 80 mK for a) device A1 and b) device A2. A moving average was applied to the data along B_z before computing the differential conductance.

6 Device overview

Figure 5 shows an overview of the nanowire device parameters. Here, L is the channel length, W is the channel width, H is the channel height, θ is the angle between the longitudinal axis of the nanowire and the direction of the magnetic field and $R(B_z = 0)$ is the device resistance at $B_z = 0$ after subtraction of the line resistance.

Device	x	L (nm)	W (nm)	H (nm)	Angle (°)	$R(B_z = 0)(\Omega)$
F10	0.27	800 ± 20	78 ± 5	130 ± 0	2 ± 3	$2.0 \cdot 10^4$
F11	0.27	600 ± 20	67 ± 5	71 ± 2	0 ± 3	$2.2 \cdot 10^4$
F12	0.27	500±20	128 ± 10	139 ± 3	11 ± 3	$1.0 \cdot 10^4$
F13	0.27	400±20	85 ± 5	128 ± 3	11 ± 3	$2.5 \cdot 10^3$
F14	0.27	400±20	77 ± 5	84 ± 1	5 ± 3	$5.8 \cdot 10^4$
A1	0.32	600±20	62 ± 8	58 ± 1	28 ± 3	$2.1 \cdot 10^3$
A2	0.32	400±20	56 ± 4	68 ± 1	0 ± 3	$3.3 \cdot 10^3$
F1	0.32	800±20	69 ± 7	39 ± 2	18 ± 3	$5.0 \cdot 10^3$
F2	0.32	1000±20	155 ± 5	155 ± 5	11 ± 3	$5.6 \cdot 10^2$
F3	0.32	800±20	91 ± 6	43 ± 4	15 ± 3	$4.4 \cdot 10^2$
B1	0.51	400±20	53 ± 8	56 ± 1	16 ± 3	$9.9 \cdot 10^2$
B2	0.51	600±20	93 ± 13	93 ± 4	5 ± 3	$8.5 \cdot 10^2$
B3	0.51	250±20	55 ± 7	62 ± 2	17 ± 3	$2.2 \cdot 10^3$
B4	0.51	400±20	95 ± 9	103 ± 2	12 ± 3	$4.7 \cdot 10^2$
B5	0.51	400±20	94 ± 11	103 ± 3	0 ± 3	$6.9 \cdot 10^2$
F4	1	400±20	89 ± 5	123 ± 3	9 ± 3	26
F5	1	1200±20	87 ± 5	115 ± 0	5 ± 3	85
F7	1	800±20	87 ± 9	106 ± 6	1 ± 3	93
F8	1	400±20	76 ± 8	102 ± 5	18 ± 3	44
F9	1	500±20	71 ± 10	80 ± 5	0 ± 3	85

Figure 5: Overview of device parameters of $Pb_{1-x}Sn_xTe$ nanowire devices discussed in this work.

7 Fast Fourier transform analysis

For the FFT spectra in Figure 2 in the main text, a 4th order polynomial fit was subtracted as background, prior to computing the Fast Fourier Transform of the data. We applied a convolution function on the raw magnetoconductance traces before subtracting the background. The power spectrum PS was obtained using $PS = 2(|y_F|/N)^2$, where y_F is the FFT data and N is the number of data points. For the analysis of the full set of devices presented in Figure 4 of the main text, we instead subtracted the mean conductance as a background. Figure 6 shows an overview of the FFT power spectra of all devices. We then applied a Gaussian filter of width 0.1 1/T centered on the frequencies h/e, $h/2e$, and $h/3e$, defined by the cross-sectional area of the nanowire, and numerically integrated to obtain the area underneath the peaks U. To obtain U_{tot}, we integrated the full spectrum before applying the Gaussian filter.

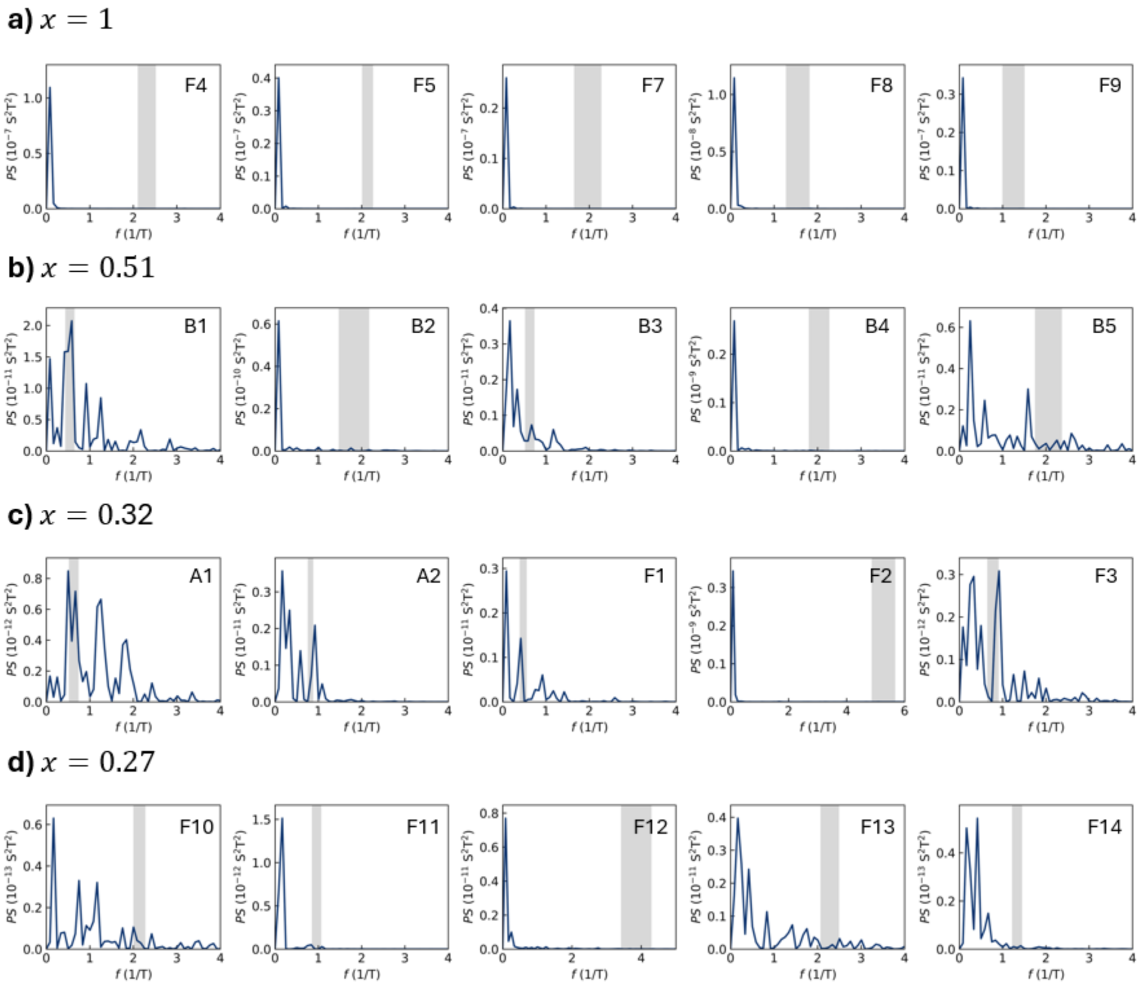

Figure 6: Overview of the FFT power spectra of the measured $Pb_{1-x}Sn_xTe$ nanowire devices with a) $x = 1$, b) $x = 0.51$, c) $x = 0.32$, d) $x = 0.27$. The spectra display PS in S^2T^2 as a function of the frequency f in 1/T. The grey regions indicate the expected frequency range for Φ_0-periodic oscillations, determined by the nanowire dimensions and associated uncertainties. The original magnetoconductance data was smoothed using a convolution function with $n = 4$, and the average conductance was subtracted as the background, prior to computing the FFT.

7.1 Background subtraction method

A fourth-order polynomial fitting method was used to subtract the slowly varying background conductance from the data in Figure 2 and Figure 3 of the main text. This approach provides a controlled means of background removal that preserves both the frequency and amplitude of oscillatory features in the spectrum. To avoid overfitting, we ensured that only the lowest frequencies corresponding to slow background variations are removed from the FFT spectrum.

For devices exhibiting oscillations, we find that the FFT results are largely insensitive to the choice of polynomial order, provided the order remains low. Figure 7 compares several background subtraction methods applied on the same dataset (device A1). Figure 7a shows the magnetoconductance and its FFT spectrum without background removal. A large peak at zero frequency is clearly observed in the FFT, corresponding to the background conductance. Figure 7b includes a first-order polynomial fit (shown in orange), which was subtracted prior to computing the FFT. This effectively removes the peak at the origin. Figure 7c shows the results for a fourth-order polynomial subtraction. The FFT spectrum is nearly identical to that in Figure 7b, indicating that the higher-order polynomial does not distort the oscillations.

As an alternative method, Figure 7d presents the numerical derivative of the magnetoconductance, a technique which has been previously used to suppress low-frequency components in similar studies, e.g. on TI nanowires [4]. This method enhances the relative strength of high-frequency components compared to low-frequency components in the FFT. We find that, although the positions of the peaks in the spectrum remains unchanged, the amplitude of the peaks is significantly distorted. Based on these comparisons, we conclude that the polynomial background subtraction method offers the most reliable approach for analyzing oscillations in the magnetoconductance data.

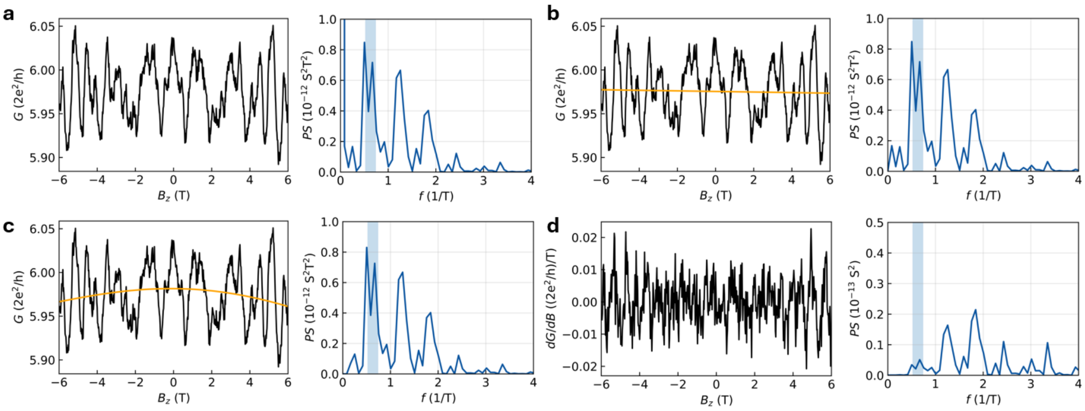

Figure 7: Comparison of different background subtraction methods on magnetoconductance data of device A1. a) Magnetoconductance trace prior to background removal including corresponding FFT power spectrum. b) Magnetoconductance trace including first-order polynomial fit (shown in orange), which was subtracted prior to computing the FFT spectrum. c) Same as b), but for a fourth-order polynomial fit. d) Numerical derivative dG/dB of the magnetoconductance, including FFT power spectrum.

8 Temperature dependence fits

Figure 8a,c shows the temperature dependence of the oscillation amplitude for devices A1 ($x = 0.32$) and B1 ($x = 0.51$), respectively. The data is fitted using Equation 1 in the main text for $m = 0.5$ and $m = 1$. For both devices, the data is best fitted with $m = 1$. Figure 8b,d shows the dependence of the coherence length on temperature, calculated using $l_\phi = CT^{-m}$, for $m = 0.5$ and $m = 1$.

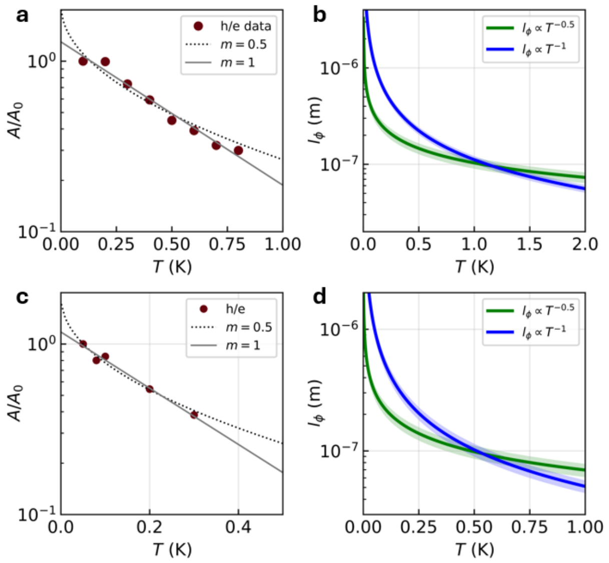

Figure 8: a) A/A_0 as a function of T, fitted using Equation 1 in the main text for different values of m, for a) $x = 0.32$ (device A1), c) $x = 0.51$ (device B1). b) l_ϕ calculated from the fits in a) as a function of temperature, including uncertainties obtained from the fitting. d) Same as b), but using the fits from c).

8.1 Additional data device A1

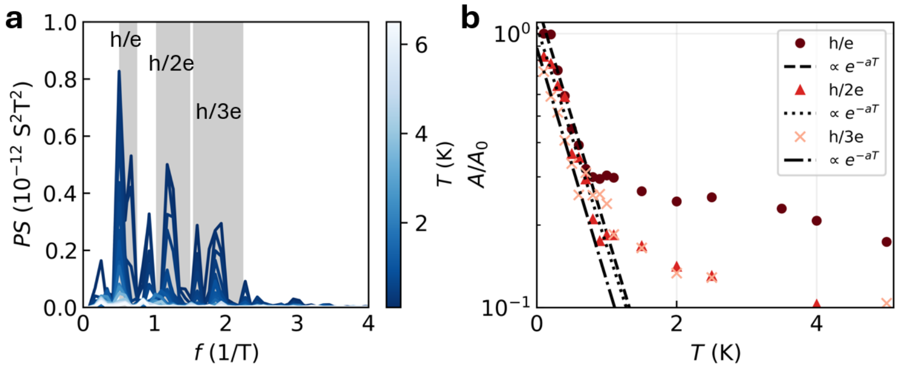

Figure 9: Temperature dependence of all harmonics for device A1 with $x = 0.32$. a) FFT power spectrum of the as a function of temperature, with the grey areas denoting the frequency range for the h/e, $h/2e$ and $h/3e$ harmonics. b) Integrated amplitudes of the h/e, $h/2e$ and $h/3e$ peaks as a function of T, fitted below $T \approx 0.7$ K with a function of the form e^{-aT}.

9 Surface-bulk ratio

$Pb_{1-x}Sn_xTe$ has a bulk carrier density n_b which scales with x according to

$$p_b(x_{Sn}) = -p_{n,PbTe} + n_{p,SnTe} \cdot e^{-\Delta E_V(1-x_{Sn})/kT}, \tag{1}$$

where $n_{n,PbTe}$ and $n_{n,PbTe}$ are the intrinsic carrier concentrations of the binaries SnTe and PbTe, and $\Delta E_V = 360$ meV is the difference in cation vacancy formation energy between SnTe and PbTe [5]. This gives $p_b \approx 2 \times 10^{20}$ for $x = 1$, $p_b \approx 1 \times 10^{19}$ for $x = 0.51$, and $p_b \approx 2 \times 10^{18}$ for $x = 0.32$. The surface state carrier density p_s of STO-covered SnTe films was measured to be $p_s = 5 \cdot 10^{14}$ cm^{-2} from Hall measurements, and $p_s = 1.3 \cdot 10^{14}$ cm^{-2} estimated from the surface band structure measured in ARPES. For SnTe films in vacuum, $p_s = 5 \cdot 10^{12}$ cm^{-2} was found, which is what we use for the calculation [6].

The total amount of bulk carriers N_b is given by

$$N_\mathrm{b} = p_\mathrm{b} D^2 L. \tag{2}$$

The total amount of surface state carriers is given by

$$N_\mathrm{s} = 4 p_\mathrm{s} D L. \tag{3}$$

The contribution of the surface state carriers to the total number of carriers is then given by

$$N_\mathrm{ss} = N_\mathrm{s}/(N_\mathrm{s} + N_\mathrm{b}) \times 100\%. \tag{4}$$

$$= 4p_\mathrm{s}/(4p_\mathrm{s} + p_\mathrm{b} D) \times 100\%. \tag{5}$$

10 Universal conductance fluctuations

To extract the UCF, we applied an Inverse Fast Fourier Transform (IFFT) on the FFT spectrum after applying a Gaussian filter with width 0.1 1/T centered around the frequencies corresponding to h/e, $h/2e$ and $h/3e$. We then subtracted this IFFT, corresponding to the periodic AB-type oscillations, from the original magnetoconductance data. The autocorrelation function $F(\Delta B_z)$ was then computed for $B_z \geq 0$ T. A cubic interpolation was applied to the data, before extracting the full-width at half-maximum of the peak centered around $\Delta B_z = 0$ T. The correlation field B_c was extracted using $F(B_c) = 1/2F(0)$.

The correlation field B_c of the conductance fluctuations can be used to determine the phase coherence length using $l_\phi = \gamma \Phi_0 / B_c d$, where d is equal to H or W, depending on the dimensions of the nanowire and the angle with respect to B_z.

For a nanowire that is parallel to the field B, d is equal to W for $W < H$, and d is equal to H for $W > H$. Figure 10a shows a schematic of a nanowire device parallel to B, and Figure 10b shows the cross section in the plane perpendicular to B. The red line indicates the loop formed by the surface states, and the light gray area indicates a loop formed by the bulk states. l_ϕ is limited by W or H, because $A = WH$ is the area defined by the largest loop that coherent bulk carriers can form in the plane perpendicular to B. For $l_\phi > W, H$, this method underestimates the value of the coherence length.

For a nanowire device at finite angle θ with B as in Figure 10c, the loops formed by coherent bulk carriers in the plane perpendicular to B are larger. Figure 10d shows the cross section of the device in Figure 10c in the plane perpendicular to B, where W_eff is the effective width in the plane parallel to B. Now, l_ϕ is limited by W_eff, because $W_\mathrm{eff} > H$.

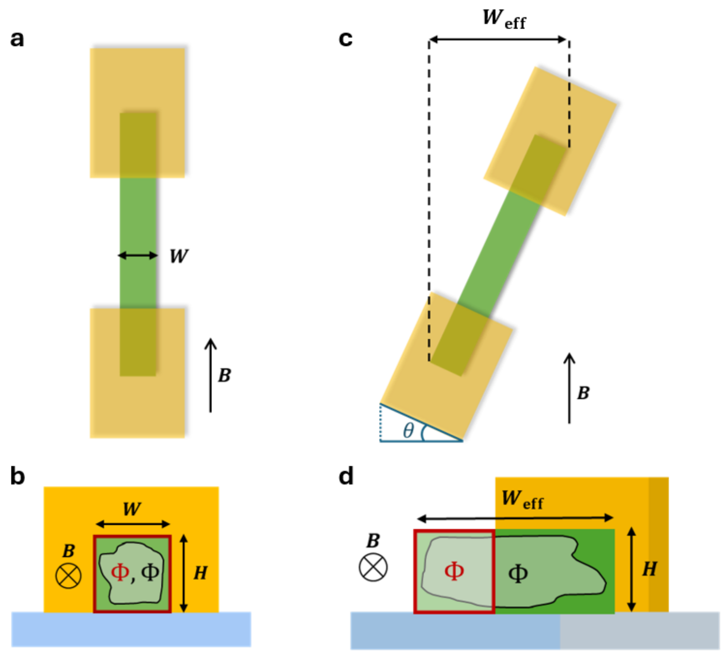

Figure 10: Schematic representation of a nanowire device. a) Top view of a device parallel to B. b) Cross-sectional front view of a), with the loop formed by the surface states indicated in red, and the loop formed by the coherent bulk states in gray. c) Top view of a device under an angle θ with respect to B. d) Same as b), but for the device in c).

For thin nanowires under an angle with the magnetic field, the smallest surface in which the phase-coherent loops exist does not always lie in the plane orthogonal to the magnetic field, see Figure 11. This is the case for devices A1 and B1, such that the extracted values for l_ϕ underestimate the coherence length in these devices. Therefore, a geometric correction was applied, based on the dimensions and angle of each device. The reported values in the main text are the corrected values, presented by $l_{\phi,\text{corr}}$ in Figure 11.

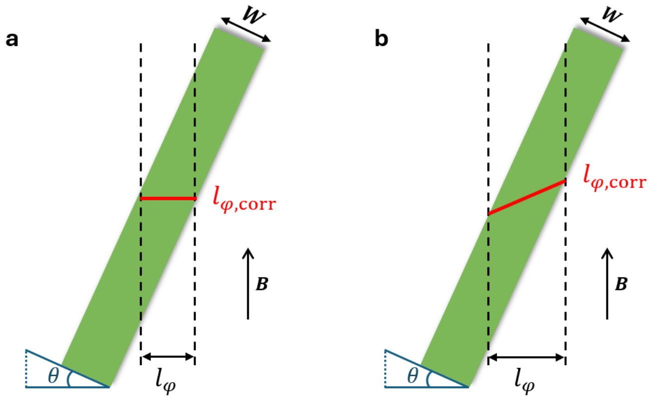

Figure 11: Schematic representation of a nanowire under an angle with the applied magnetic field. The surface in which phase-coherent loops exist, based on l_ϕ, is given by a red line. a) This surface is orthogonal to the magnetic field direction, such that $l_{\phi,\text{corr}} = l_\phi$. b) This surface is not orthogonal to the magnetic field direction, such that $l_{\phi,\text{corr}} > l_\phi$.

10.1 Additional data UCF temperature dependence

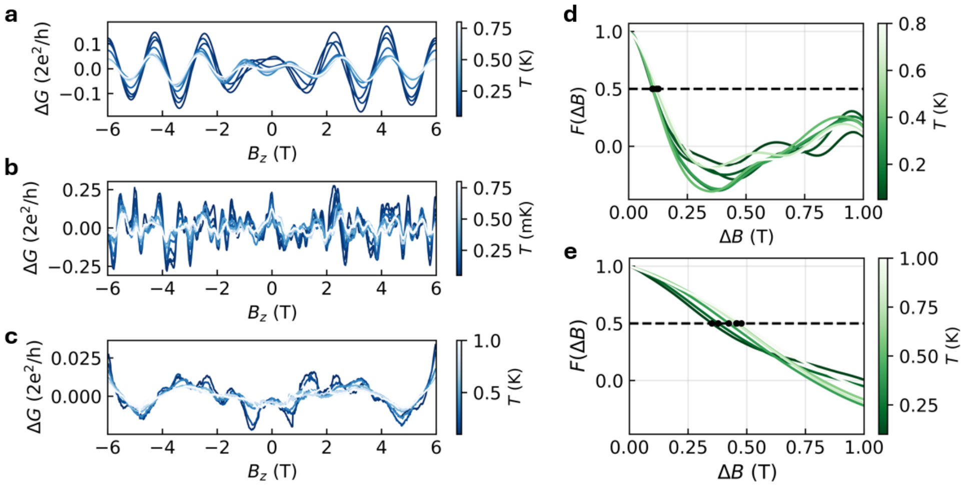

Figure 12: a) Inverse FFT of the h/e peak for different temperatures for $x = 0.51$ (device B1). b) The magnetoconductance for different temperatures of the same device, with the IFFT data from a) subtracted. c) Magnetoconductance for different temperatures for $x = 0.27$ (device F11). The $T = 300$ mK data was removed as an outlier. d) Autocorrelation function $F(\Delta B)$ of the data in d), for positive B_z. The black dots indicate the correlation field B_c. e) Same as d), but for the data in c).

11 Additional TEM/EDX data on FIBCUT of device B1

Figure 13 shows additional TEM and EDX data on device B1. Figure 13a shows a BF-STEM image of the entire device, including the four contacts. Figure 13b shows an elemental EDX mapping of Te, Au and Si. No intermixing is observed between the nanowire and the contacts or the substrate. Figure 13c shows HAADF STEM of the middle channel between the two inner probes, with blue dots indicating the locations for the EDX quantification shown in Figure 13f. The line profile was acquired by measuring 20s per blue dot. The composition remains constant over the entire length of the channel. Figure 13d shows a HR TEM image zoomed in on the center part of the middle channel. The center part of the nanowire is mono-crystalline, with a layer of approx. 5 nm on the top and bottom consisting of native oxide. Additionally, some amorphous regions are observed close to the native oxide. The formation of amorphous regions has not been observed before in TEM studies of $Pb_{1-x}Sn_xTe$ nanowires that were not milled by FIB. Furthermore, the parts of the nanowire covered by the Cr/Au contacts are perfectly crystalline. Therefore, we conclude that the formation of the amorphous regions is likely induced by the FIB during milling. Figure 13e shows the FFT analysis of the mono-crystalline center part of the nanowire in Figure 13d.

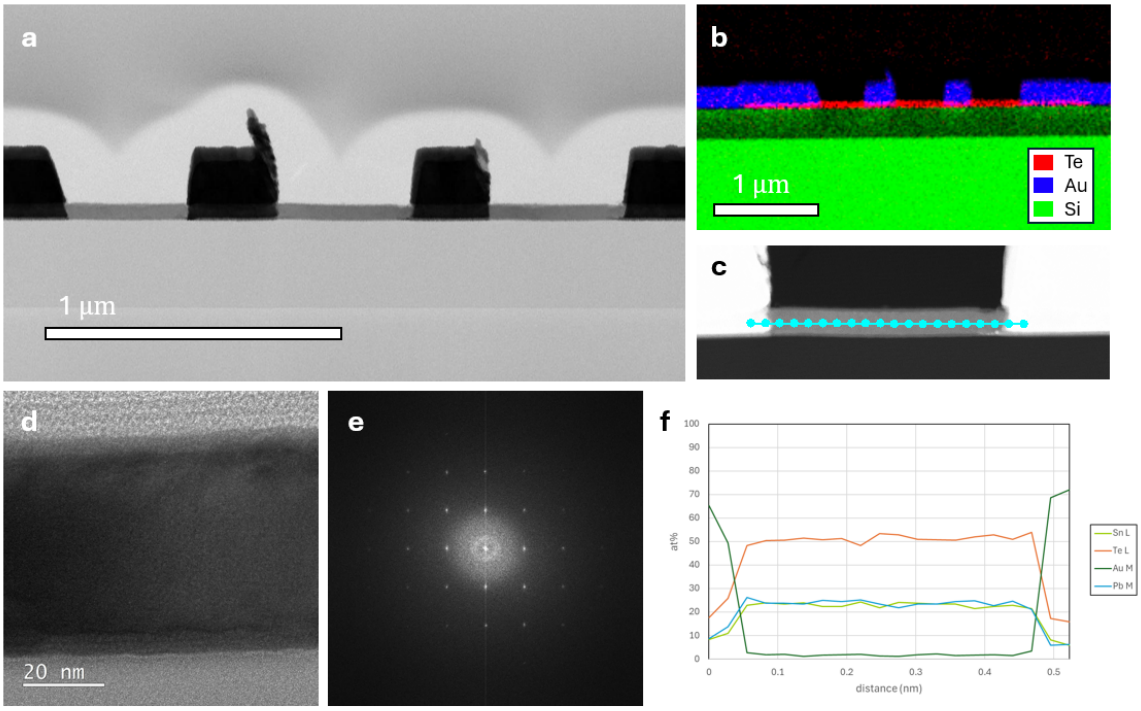

Figure 13: TEM and EDX data on a FIBCUT of device B1. a) BF-STEM image. b) EDX elemental mapping of Te, Au and Si. c) HAADF STEM of the channel between the two inner probes. The light-blue dots indicate the locations of the EDX quantification in f). d) High-resolution TEM of the channel between the two inner probes. e) FFT analysis of the interior of the nanowire in d). f) EDX compositional line profile (quantified), acquired by measuring 20s per point along the line indicated in c).

References

[1] M. G. C. Mientjes, X. Guan, P. J. H. Lueb, M. A. Verheijen, and E. P. A. M. Bakkers, “Catalyst-free MBE growth of PbSnTe nanowires with tunable aspect ratio,” *Nanotechnology*, vol. 35, p. 325602, may 2024.

[2] Stiles, P. J., “Tunneling in IV-VI compounds,” *J. Phys. Colloques*, vol. 29, pp. C4–105–C4–113, 1968.

[3] “CRC Handbook of Chemistry and Physics, 88th ed Editor-in-Chief: David R. Lide (National Institute of Standards and Technology) CRC Press/Taylor & Francis Group: Boca Raton, FL. 2007. 2640 pp. ISBN 0-8493-0488-1.,” *Journal of the American Chemical Society*, vol. 130, no. 1, pp. 382–382, 2008.

[4] H. Peng, K. Lai, D. Kong, S. Meister, Y. Chen, X. Qi, S. Zhang, X. Shen, and Y. Cui, “Aharonov–Bohm interference in topological insulator nanoribbons,” *Nature Materials*, vol. 9, pp. 225–229, dec 2010.

[5] V. V. Volobuev, P. S. Mandal, M. Galicka, O. Caha, J. Sánchez-Barriga, D. Di Sante, A. Varykhalov, A. Khiar, S. Picozzi, G. Bauer, P. Kacman, R. Buczko, O. Rader, and G. Springholz, “Giant Rashba Splitting in $Pb_{1-x}Sn_xTe$ (111) Topological Crystalline Insulator Films Controlled by Bi Doping in the Bulk,” *Advanced Materials*, vol. 29, no. 3, p. 1604185, 2017.

[6] K. Zou, S. D. Albright, O. E. Dagdeviren, M. D. Morales-Acosta, G. H. Simon, C. Zhou, S. Mandal, S. Ismail-Beigi, U. D. Schwarz, E. I. Altman, F. J. Walker, and C. H. Ahn, “Revealing surface-state transport in ultrathin topological crystalline insulator SnTe films,” *APL Materials*, vol. 7, p. 051106, 05 2019.
